# Multifocal Osteoarticular Infection by Methicillin-Sensitive Staphylococcus aureus in the Neonatal Period: A Diagnostic Challenge

**DOI:** 10.7759/cureus.103123

**Published:** 2026-02-06

**Authors:** Filipe Ramos, Joana Ovídio, Joana Arcangelo, João L Campagnolo, Beatriz Sousa Nunes, Catarina Gouveia

**Affiliations:** 1 Pediatric Orthopaedics, Unidade Local de Saúde São José - Hospital Dona Estefânia, Lisbon, PRT; 2 Orthopaedics and Traumatology, Unidade Local de Saúde Santa Maria, Lisbon, PRT; 3 Pediatrics, Unidade Local de Saúde São José - Hospital Dona Estefânia, Lisbon, PRT; 4 Pediatric Infectious Diseases, Unidade Local de Saúde São José - Hospital Dona Estefânia, Lisbon, PRT

**Keywords:** emergency orthopedics, hematogenous spread, methicillin-sensitive staphylococcus aureus, neonatal infection, osteoarticular infection, pediatrics emergency, wrist septic arthritis

## Abstract

We report the case of a 15-day-old neonate presenting with multifocal osteoarticular infection, initially manifesting as septic arthritis of the right wrist, which rapidly progressed to involve multiple sites, including the right deltoid and left hip. The etiological agent was identified as methicillin-sensitive *Staphylococcus aureus* (MSSA). This case is notable for its multifocality, the severity of the clinical course in the neonatal period, and the association with a prior history of neonatal admission for severe hypernatremic dehydration.

## Introduction

Septic arthritis and osteomyelitis in the neonatal period, collectively referred to as osteoarticular infections (OAI), are rare but severe conditions involving the infection of joints, bones, or both. Neonates are uniquely vulnerable to these infections due to their immature immune systems and specific bone vascularization, where transphyseal vessels allow for the easy spread of infection between the metaphysis and the joint space [1].

These infections present a diagnostic challenge due to the scarcity of signs and symptoms, which can lead to delayed recognition [2,3].

Multifocal disease is most commonly caused by *Staphylococcus aureus* and is a particularly severe presentation, representing systemic involvement and increased risk of progression to multi-organ dysfunction. Staphylococci can produce a profound variety of clinical syndromes in newborn infants, including skin and soft tissue abscesses and invasive infections such as bacteremia, sepsis, pneumonia, meningitis, osteomyelitis, and septic arthritis [4,5].

These infections are associated with a worse prognosis and morbidity [2], potentially causing devastating long-term orthopedic sequelae, such as premature growth plate closure, limb length discrepancy, osteonecrosis, and permanent functional impairment [3]. This condition requires rapid diagnosis and aggressive medico-surgical treatment to limit disease dissemination and prevent debilitating long-term sequelae.

## Case presentation

Background and clinical presentation

**Figure 1:**
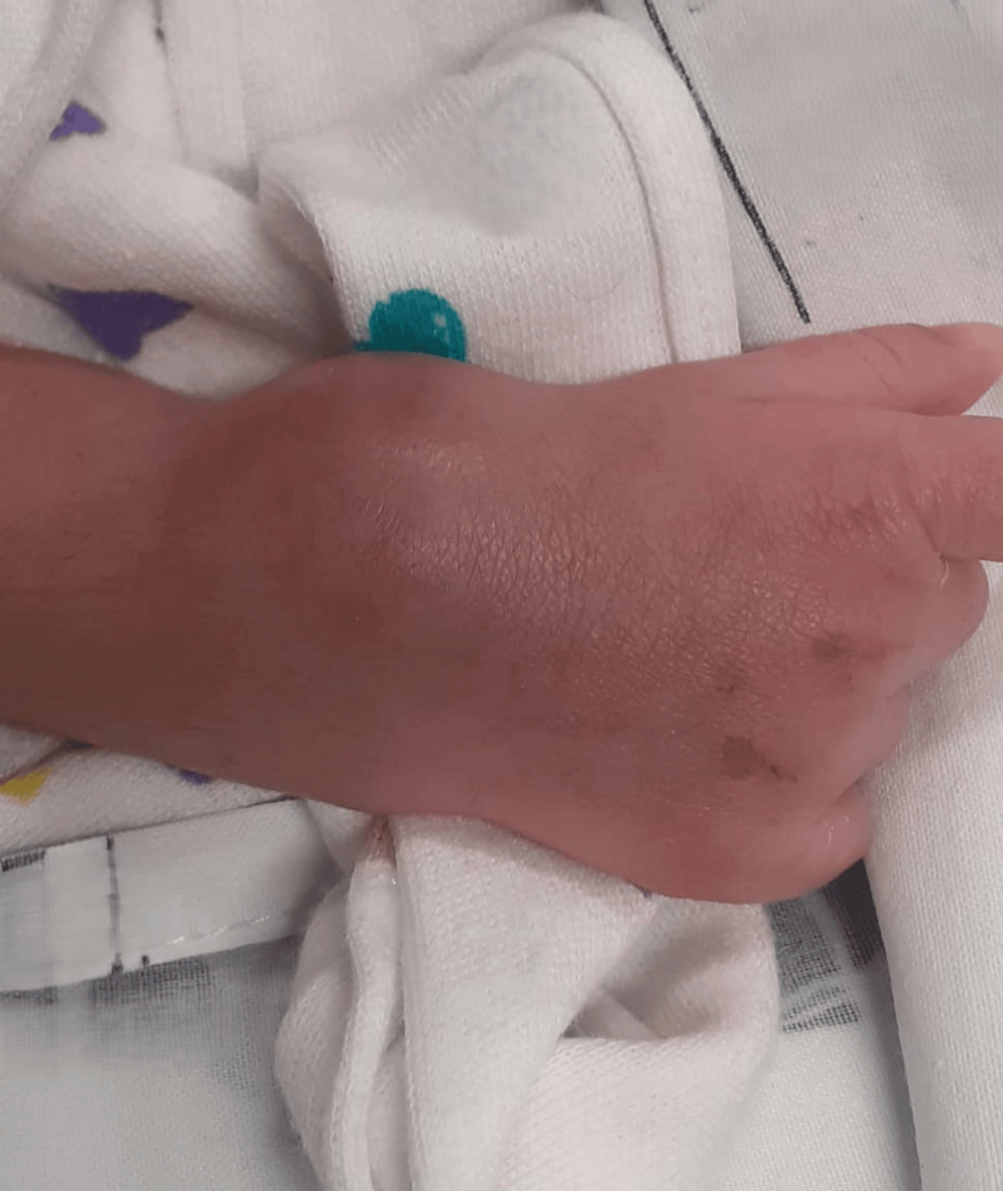
Clinical appearance of the right wrist at admission. Edema and erythema consistent with an underlying abscess and associated septic arthritis.

A full-term male neonate (37 weeks of gestation) was born via cesarean section due to breech presentation, with a birth weight of 2,840 g. Pregnancy was adequately supervised, and maternal Group B *Streptococcus *screening was negative. The patient had been previously hospitalized in a neonatal unit between day 5 and day 9 of life for severe hypernatremic dehydration (17% weight loss, serum sodium 158 mmol/L), associated with hypoglycemia and hyperbilirubinemia, requiring intravenous hydration and phototherapy. At 15 days of age, he presented to the emergency department with a three-day history of painful swelling and erythema of the right wrist. A febrile peak of 38°C was recorded during initial evaluation. On examination, the neonate appeared otherwise well but exhibited pseudoparalysis of the right upper limb with localized pain and edema of the right wrist (Figure 1).

Diagnostic workup and management

**Figure 2:**
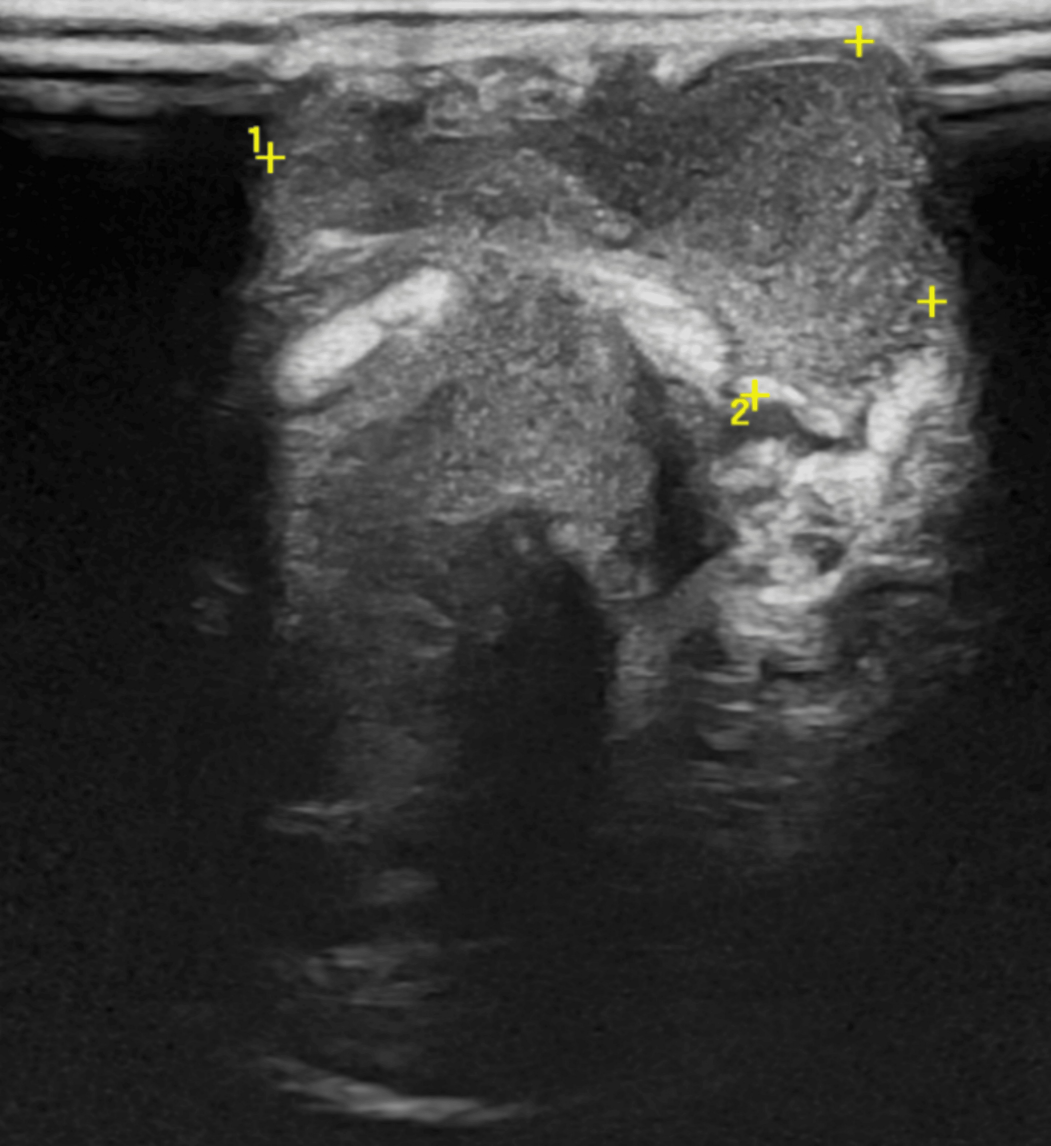
Right wrist ultrasound showing soft tissue abscess Ultrasound image demonstrating a heterogeneous collection located within the subcutaneous tissue and deep planes. Yellow calipers and numbers 1 and 2 demarcate the boundaries of the lesion (18 x 16 x 9 millimeters).

**Figure 3:**
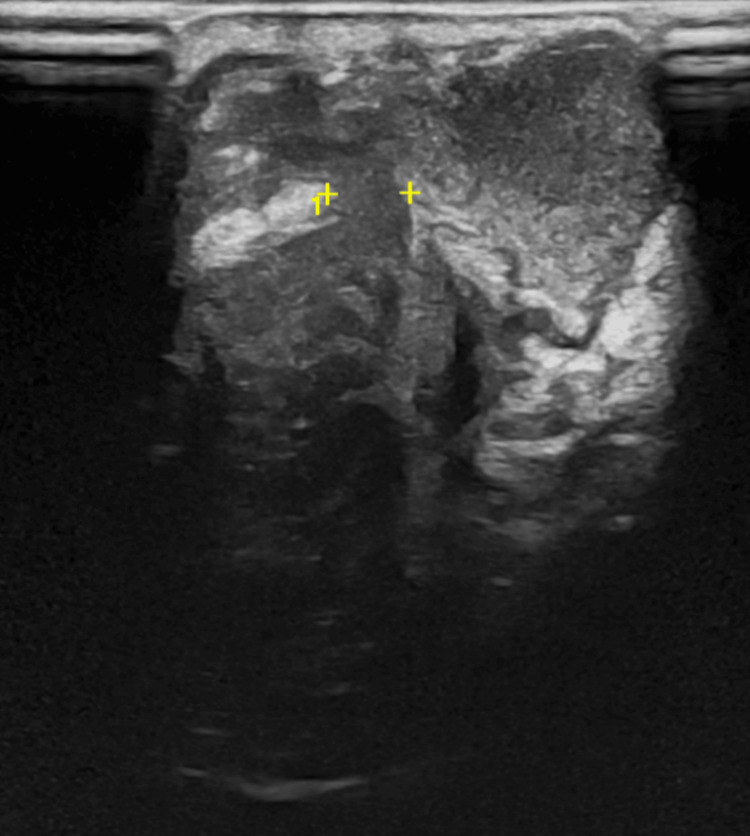
Right wrist ultrasound showing intra-articular extension. Ultrasound view highlighting the tract of the abscess collection extending through the deep planes into the joint and echogenic material filling the joint space.

Laboratory studies on admission revealed leukocytosis (23,600/µL; reference interval: 5,000-19,000/µL) with neutrophilia (14,700/µL, 62%) and elevated C-reactive protein (CRP) of 47.8 mg/L (reference value: <5.0 mg/L). Joint ultrasonography of the right wrist demonstrated a cutaneous abscess communicating with the radiocarpal joint, containing heterogeneous intra-articular debris (Figures 2-3).

Given the diagnosis of a soft tissue abscess with articular extension, consistent with septic arthritis of the wrist, the patient was transferred to our institution, a tertiary pediatric hospital, and underwent urgent surgical drainage of the abscess, arthrocentesis, and irrigation of the radiocarpal joint, followed by initiation of intravenous antibiotic therapy.

Synovial fluid cultures isolated methicillin-sensitive *Staphylococcus aureus *(MSSA), susceptible to oxacillin and gentamicin. Polymerase chain reaction testing for Panton-Valentine leukocidin was negative. Serial blood cultures were also negative.

**Figure 4:**
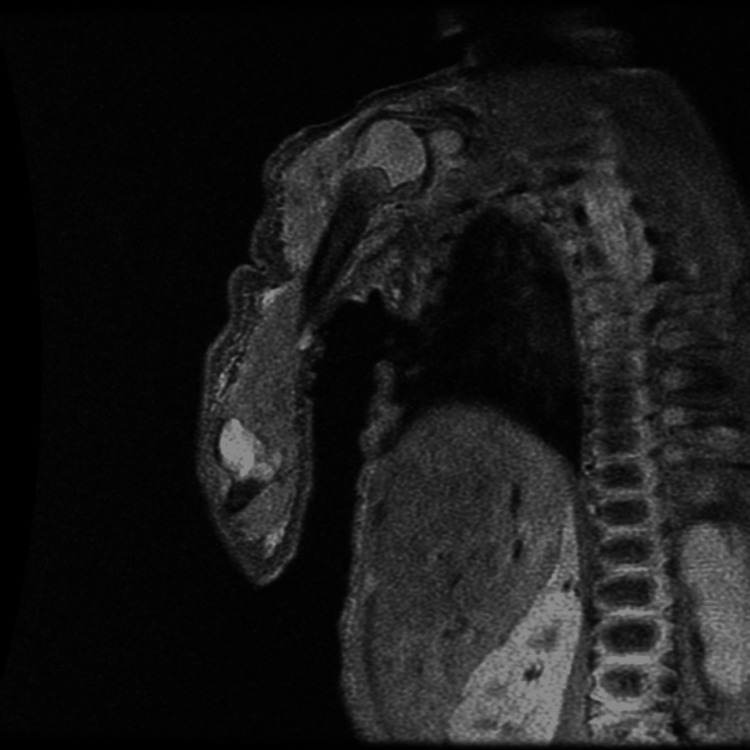
Coronal T2-weighted (STIR) right upper limb MRI demonstrating extensive inflammatory process on the shoulder. The image reveals marked, diffuse high signal intensity involving the soft tissues of the shoulder region, indicative of edema and myositis. Intra-articular hyperintensity is also noted within the glenohumeral joint, consistent with effusion.

Approximately 48 hours later, localized inflammatory signs developed on the right shoulder. Ultrasonography confirmed a fluid collection within the deltoid muscle, which was managed by needle aspiration. Cultures again yielded MSSA. Subsequent magnetic resonance imaging (MRI) of the right upper limb demonstrated mild glenohumeral joint effusion (maximum thickness of 3 mm) and deltoid myositis, without evidence of osteomyelitis of the proximal humerus (Figure 4).

**Figure 5:**
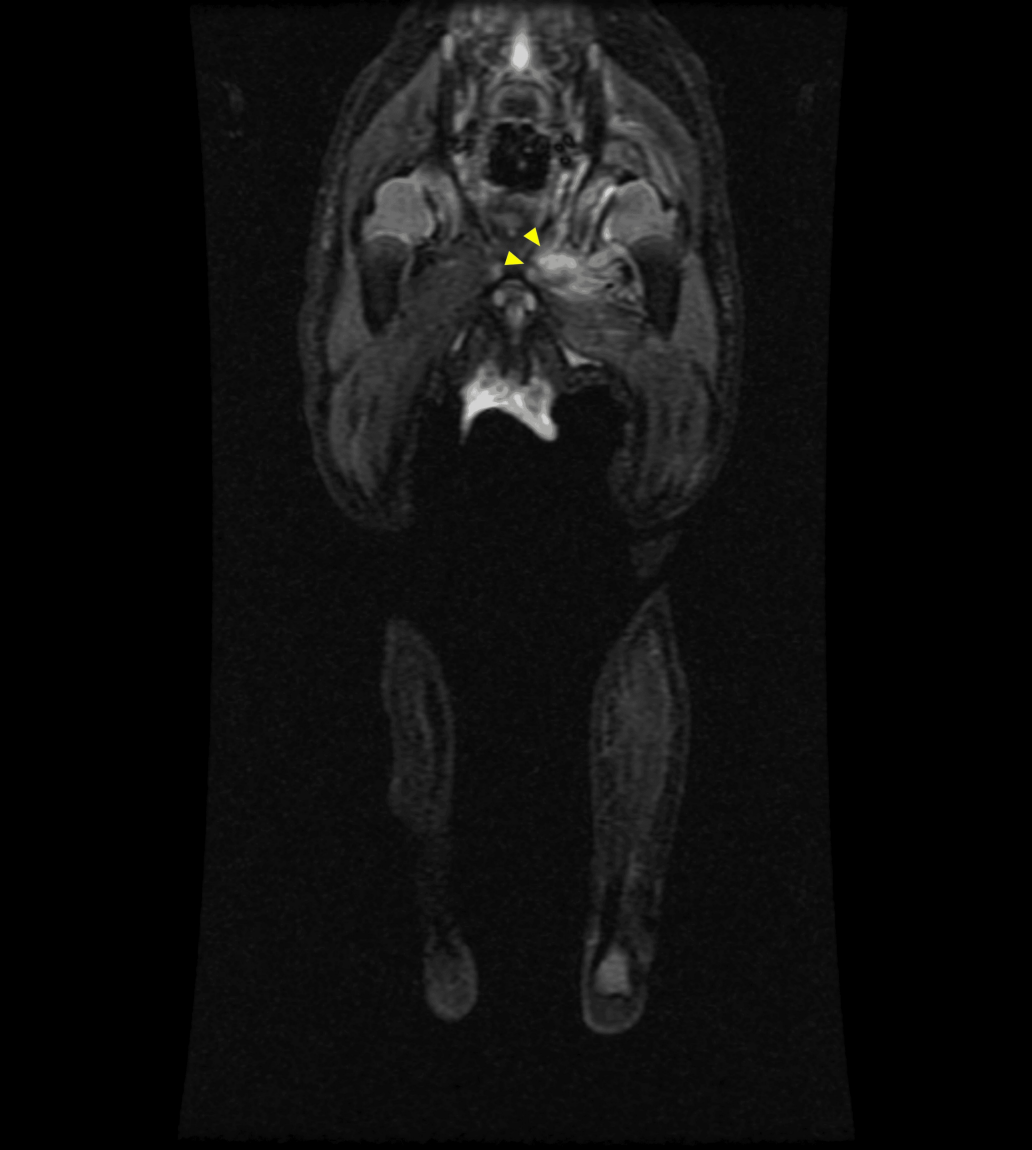
Coronal T2-weighted MRI of the left hip. Marked high signal intensity is observed involving the left pectineus and adductor musculature, suggestive of myositis (yellow arrowheads).

**Figure 6:**
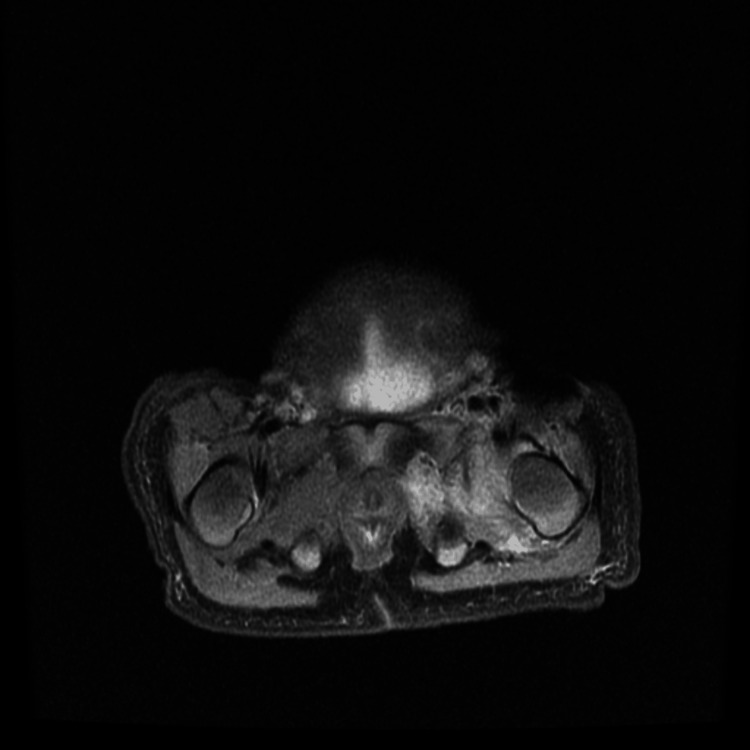
Axial T2-weighted MRI of the left hip. Axial image showing joint effusion in the left hip joint and inflammation of the surrounding soft tissues.

Three days later, reduced spontaneous movement of the left lower limb was noted, accompanied by edema of the left thigh and ankle. Initial ultrasonography revealed diffuse subcutaneous edema of the thigh without a defined abscess. MRI was therefore performed to further assess disease extent and exclude deep abscess or joint involvement. Imaging demonstrated a small left coxofemoral joint effusion associated with myositis/pyomyositis and a 5 × 3 mm intramuscular abscess within the adductor-pectineus muscle group, along with surrounding cellulitis of the thigh. There was no evidence of osteomyelitis (Figures 5-6).

At this stage, the patient was afebrile and showed clear clinical improvement, with a marked decline in inflammatory markers (CRP decreased from 22.8 to 3.1 mg/L). Given the favorable clinical and biochemical evolution under appropriate intravenous antibiotic therapy, the small joint effusion, and the absence of systemic toxicity, conservative management with close clinical and imaging surveillance was elected.

Two days later, CRP further declined to 0.6 mg/L, with continued reduction in erythrocyte sedimentation rate (51 to 40 mm/h). Repeat hip ultrasonography showed persistent synovitis and myositis, a thin layer of joint effusion, no abscesses, and a reduced alpha angle of 54° (Graf type IIA). Transthoracic echocardiography was performed to exclude infective endocarditis and revealed no structural abnormalities or valvular vegetations.

Treatment and outcome

Intravenous antibiotic therapy was initiated with flucloxacillin (200 mg/kg/day) and gentamicin (4 mg/kg/day). Gentamicin was discontinued after four days, following exclusion of cardiac involvement. The patient showed progressive resolution of local inflammatory signs and improvement in limb mobility. After 21 days of intravenous therapy, treatment was transitioned to oral flucloxacillin, and the patient was discharged. In total, he completed six weeks of antibiotic therapy. Orthopedic follow-up identified left hip dislocation (Graf type III), treated with a Pavlik harness, with progressive improvement and a concentric hip joint on follow-up imaging at one year.

A timeline showing key findings and interventions is shown in Table 1.

**Table 1:** Clinical timeline, diagnostic findings, and therapeutic interventions from admission to discharge and follow-up. AB: antibiotic; CRP: C-reactive protein; MRI: magnetic resonance imaging; OR: operating room; US: ultrasound.

Day of Illness	Findings and therapeutics
Day 1 (15 days old)	Three-day history of right wrist swelling
	Clinical signs: fever (single febrile peak), edema, pain, pseudoparalysis
	LAB: Leukocytosis, neutrophilia, CRP 47.8 mg/L, ESR 51 mm/h
	US: Wrist abscess with joint communication
	OR: drainage and joint lavage
	Starts AB: Flucloxacillin + gentamicin
Day 2-3	Inflammatory signs on the right shoulder
	US: Intramuscular abscess in the right deltoid
	Requested upper limb MRI
	OR: Deltoid abscess aspiration (2cc of purulent fluid)
Day 4	Echocardiogram negative for vegetations
	Stops gentamicin
Day 5	MRI: Mild glenohumeral effusion (3mm) and deltoid myositis. No signs of osteomyelitis or organized abscess on MRI
Day 6	Left lower limb edema, reduction of spontaneous movement
	Requested US
Day 9	Labs: CRP 22.8 mg/L
Day 10	Ultrasound: Subcutaneous edema in the thigh; trace fluid in the ankle (1.4mm).
	Requested lower limb MRI
Day 13	MRI: Mild hip joint effusion, myositis in the obturator, quadratus femoris, and adductors.
	CRP dropped to 3.1 mg/L
	Orthopedics: Clinical monitoring
Day 16	Clinical improvement in spontaneous movement and edema
	Reduction in inflammatory markers (CRP 0.6 mg/L and ESR 40mm/h)
Day 19	Ultrasound: Hip joint effusion + signs of dysplasia
	Exam: Spontaneous movement
	Orthopedics: Medical therapy, close monitoring
Day 20	Switches IV flucloxacillin to oral
Day 22	Sustained clinical improvement
	Negative CRP
	Discharged from hospital. Scheduled Pediatric Infectious Diseases and Orthopedic follow-up
Day 42	Terminates oral flucloxacillin (total six weeks)

## Discussion

Neonatal osteoarticular infection (OAI) remains a diagnostic challenge due to its often subtle initial clinical signs, rapid progression, and high risk for functional sequelae, if not promptly recognized and treated [2,6]. This case is particularly instructive due to its multifocal nature, which has been reported to occur in 27-50% of neonatal cases [7], frequently related to hematogenous dissemination from a primary focus or cutaneous entry point.

The pathophysiology of OAI in neonates differs from that in older children [6,7]. Persistence of transphyseal vessels allows infection to cross the growth plate, facilitating the coexistence of osteomyelitis and septic arthritis within the same anatomical segment [1]. In the present case, the virulence of MSSA was evidenced by the rapid progression from localized wrist infection to distant muscular and articular involvement, including the deltoid region and hip. Isolation of this pathogen should prompt a high index of suspicion for multifocal disease, even in the absence of clinical signs at other sites. In this case, serial blood cultures remained negative, which is consistent with existing literature reporting that blood cultures are negative in up to 70% of neonatal OAI cases [2,8,9]. This highlights the necessity of obtaining direct samples from the infectious foci, such as synovial fluid or abscess aspirates, to ensure pathogen identification and targeted antibiotic therapy.

The prognosis for neonatal OAI is significantly more unfavorable than in older infants. A retrospective study of infants under three months of age in Parisian hospitals reported late orthopedic complications in 11.8% of patients followed for more than one year [3]. However, the 48% follow-up rate in that series suggests that the true incidence of sequelae might be even higher due to potential loss-to-follow-up bias. Locally, our team’s previous research confirms this trend, showing that OAI in infants ≤3 months is associated with longer antibiotic courses and a significantly higher rate of sequelae compared to older children (17.4% vs 3.2%, P = 0.002) [2]. These findings underscore the critical importance of prolonged orthopedic surveillance in this vulnerable population.

We hypothesize that the preceding episode of severe hypernatremic dehydration might have been a contributing factor to disease dissemination. Physiological stress in neonates may exacerbate the intrinsic immaturity of the neonatal immune response, potentially impairing epithelial barrier integrity and facilitating bacterial translocation and invasive infection [10,11]. However, it is equally important to consider that prior hospitalization in a neonatal intensive care unit, with exposure to invasive procedures, is a well-established risk factor for colonization and subsequent invasive infection by *Staphylococcus aureus* [12,13].

Imaging played a central role in diagnosis and management. Ultrasonography allowed rapid identification of the wrist abscess with articular communication, enabling timely surgical intervention. MRI was essential for defining disease extent, identifying clinically occult lesions, and guiding therapeutic decision-making. In cases of suspected multifocal or deep-seated neonatal infection, MRI remains the gold standard imaging modality.

Urgent surgical drainage of the wrist was important given the clear communication between the abscess and the radiocarpal joint. Early arthrocentesis and irrigation reduced bacterial load, relieved intracapsular pressure, and minimized the risk of irreversible cartilage and growth plate damage.

Management of hip involvement required individualized clinical judgment. The minimal joint effusion, absence of osteomyelitis, rapid normalization of inflammatory markers, and favorable clinical response supported the diagnosis of reactive arthritis and a conservative approach with close surveillance. Nevertheless, the threshold for surgical intervention must remain low, as delayed treatment of true septic arthritis of the hip can lead to long-term sequelae. Breech presentation is a well-established risk factor for hip dysplasia. In this case, the potential contribution of the infectious process cannot be entirely excluded, but its role appears to be limited, and the positive evolution is more consistent with an underlying developmental condition.

## Conclusions

Multifocal osteoarticular infection caused by *Staphylococcus aureus* in the neonatal period represents a severe and potentially underdiagnosed condition, associated with rapid dissemination and a high risk of long-term sequelae. A systematic search for additional infectious foci is essential whenever this pathogen is isolated from a neonatal osteoarticular site.

Imaging plays a central role in diagnosis and management, with magnetic resonance imaging being particularly valuable for detecting clinically occult joint and soft tissue involvement.

Aggressive medical and surgical treatment strategies should always be considered and individualized to prevent irreversible joint damage and functional impairment.
